# s_±_ pairing near a Lifshitz transition

**DOI:** 10.1038/srep32078

**Published:** 2016-08-26

**Authors:** Vivek Mishra, Douglas J. Scalapino, Thomas A. Maier

**Affiliations:** 1 Joint Institute of Computational Sciences, University of Tennessee, Knoxville, TN-37996, USA; 2 Department of Physics, University of California, Santa Barbara, CA-93106, USA; 3 Computer Science and Mathematics Division & Center for Nanophase Materials Sciences, Oak Ridge National Laboratory, Oak Ridge, TN-37831, USA

## Abstract

Observations of robust superconductivity in some of the iron based superconductors in the vicinity of a Lifshitz point where a spin density wave instability is suppressed as the hole band drops below the Fermi energy raise questions for spin-fluctuation theories. Here we discuss spin-fluctuation pairing for a bilayer Hubbard model, which goes through such a Lifshitz transition. We find s_±_ pairing with a transition temperature that peaks beyond the Lifshitz point and a gap function that has essentially the same magnitude but opposite sign on the incipient hole band as it does on the electron band that has a Fermi surface.

The microscopic mechanism of pairing that gives rise to superconductivity in the iron based superconductors remains an unsettled issue^1^. Spin-fluctuation mediated pairing^2^,^3^,^4^, in which electrons form pairs by exchanging virtual S = 1 particle hole excitations, is a leading candidate mechanism, since superconductivity appears near the onset of a magnetic phase. However, this picture relies on the nesting properties of the electronic band structure with both hole and electron Fermi surface pockets present and the absence of hole pockets in some of the iron based superconductors^5^,^6^,^7^,^8^,^9^,^10^,^11^ has challenged these theories. In these systems, the hole like band drops below the Fermi energy after a Lifshitz transition^12^,^13^. Nevertheless, pairing remains strong, as evidenced *e*.*g*. by the high Tc superconductivity reported in mono-layer FeSe films grown on SrTiO_3_^9^,^10^,^11^,^14^. Scanning tunneling microscopy experiments^12^ as well as ARPES measurements^11^,^13^ on these FeSe mono-layers find that there are no hole pockets. Furthermore, the ARPES measurements of the variation of the gap magnitude around the electron pockets^13^ makes the possibility of d-wave pairing, arising from pair scattering between the electron pockets, unlikely. However, these experiments also report the existence of an incipient hole band laying 50 to 100 meV below the Fermi energy, implying that the system is just beyond a Lifshitz transition^15^ where the hole Fermi surface has disappeared. In addition, photoemission measurements find evidence that superconductivity occurs in the monolayer FeSe film, when SDW order is suppressed by electron doping^11^ and density functional theory calculations^16^ predict that in the absence of electron doping, the ground state of the mono-layer FeSe film would have SDW order. Thus, it appears that superconductivity is induced in the FeSe mono-layer when the SDW order is suppressed by a Lifshitz transition arising from electron doping or strain^11^. Motivated by these results, we have investigated the suppression of SDW order and the onset of superconductivity near a Lifshitz transition in a two-layer Hubbard model. This model was previously shown to have both *s*_±_ and *d*-wave pairing depending upon the strength of the interlayer hopping^17^. Here using this model, we show that spin-fluctuation scattering of pairs between an electron and an incipient hole band can lead to *s*_±_ pairing for a system that has undergone a Lifshitz transition.

## Results

The Hamiltonian for the two layer Hubbard model that we study is


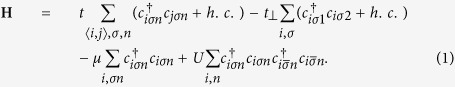


**Figure 1:**
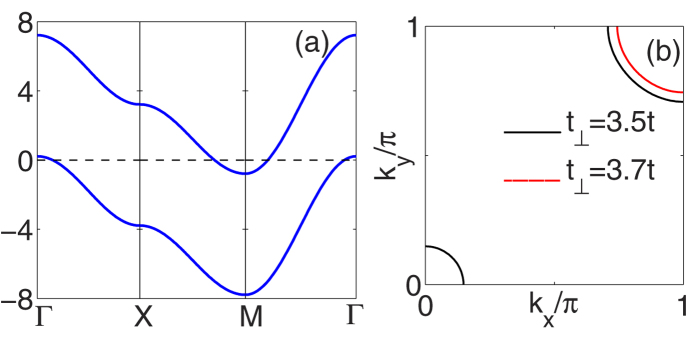
(**a**) The *k*_*z*_ = 0 and *π* energy bands of the two layer Hubbard model plotted along (0, 0) to (*π*, 0) to (*π*, *π*) to (0, 0) with *t*_⊥_ = 3.5*t* and the chemical potential *μ* adjusted for a site filling 〈*n*〉 = 1.05. (**b**) The Fermi surface for 〈*n*〉 = 1.05 with *t*_⊥_ = 3.5*t* (solid black) and *t*_⊥_ = 3.7*t* (red).

Here 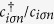 creates/annihilates a fermion with spin *σ* on the *n*^*th*^ layer (n = 1 or 2). The intralayer hoping is *t*, the interlayer hoping is *t*_⊥_ and *μ* is the chemical potential. The band structure for this model is,


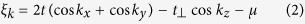


with *t*_⊥_/*t* = 3.5 and *μ* set so that the site filling 〈*n*〉 = 1.05 is shown in Fig. 1(a). If the filling is kept constant as *t*_⊥_/*t* is increased, the system has a Lifshitz transition such that for *t*_⊥_ > 3.67 the hole Fermi surface at the Γ point disappears as illustrated in Fig. 1(b). We are interested in studying the pairing for parameters such that the spin density wave (SDW) instability is suppressed by this Lifshitz transition.

In a random phase approximation (RPA) the spin susceptibility is given by


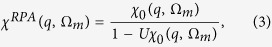


with


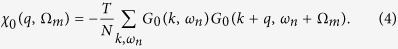


**Figure 2:**
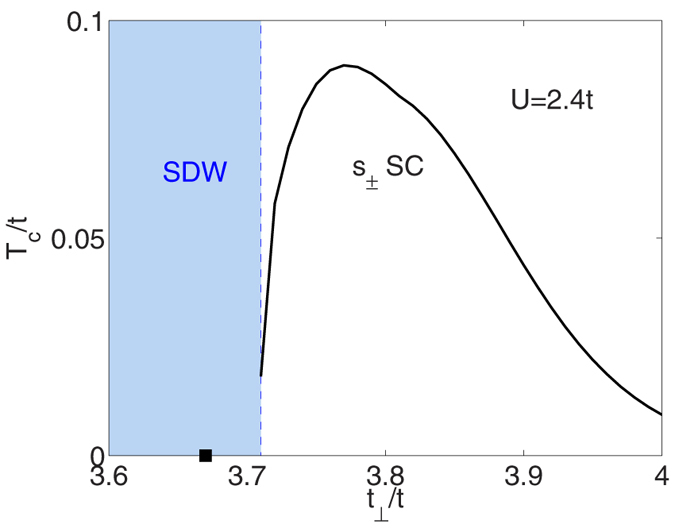
The s_±_ superconducting transition temperature *T*_*c*_ versus *t*_⊥_/*t* for 〈*n*〉 = 1.05 and *U* = 2.4*t*. The Lifshitz point is denoted by a filled square on the *t*_⊥_/*t* axis and the *t*_⊥_/*t* value where the RPA evaluated SDW instability ends by a vertical dashed line with light blue shading to the left of it.

Here *T* is the temperature, *G*_0_(*k*, *ω*_*n*_) = (*iω*_*n*_ − *ξ*_*k*_)^−1^ and *ω*_*n*_ = (2*n* + 1)*πT* and Ω_*m*_ = 2*mπT* are the usual fermionic and bosonic Matsubara frequencies. For a fixed filling, as *t*_⊥_/*t* is increased and the Lifshitz transition is approached, *χ*_0_ which peaks near wavevector (*π*, *π*, *π*), decreases. For 〈*n*〉 = 1.05, we take *U* = 2.4*t* so that the SDW instability determined from Eq. (3) is suppressed by the Lifshitz transition as shown in Fig. 2. With this suppression of the SDW order, one can imagine that superconductivity may appear following the usual paradigm. However, the Lifshitz transition that has suppressed the SDW instability can also lead to a suppression of the s_±_ pairing associated with the scattering of pairs between the electron Fermi surface and the incipient hole band. For a fixed pairing strength, *T*_*c*_ decreases as the hole band moves below the Fermi energy^18^.

To explore this, we solve the Bethe-Salpeter equation


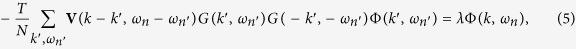


and determine *T*_*c*_ from the temperature at which the leading eigenvalue of Eq. (5) goes to 1. Here we use a spin-fluctuation mediated interaction^19^,


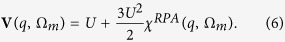


Here the first term in the effective interaction is the bare interaction *U* which is momentum independent. The effect of this term is small due to the sign change of the gap between the two bands. In Eq. (5) we set *G*(*k*, *ω*_*n*_) = [*iω*_*n*_ − *ξ*_*k*_ − Σ(*k*, *ω*_*n*_)]^−1^ with


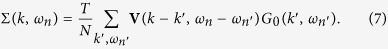


Note that we keep the Fermi surface unchanged in the dressed Green’s function. For 〈*n*〉 = 1.05 and *U* = 2.4*t*, the resulting value of *T*_*c*_, interpolated from the temperature at which *λ* crosses 1, is plotted in Fig. 2 as a function of *t*_⊥_/*t*. As shown in this figure, after the SDW instability is suppressed by the Lifshitz transition, a pairing transition occurs at a *T*_*c*_ which peaks as *t*_⊥_/*t* increases and then falls off as the hole band is pushed further below the Fermi energy.

**Figure 3:**
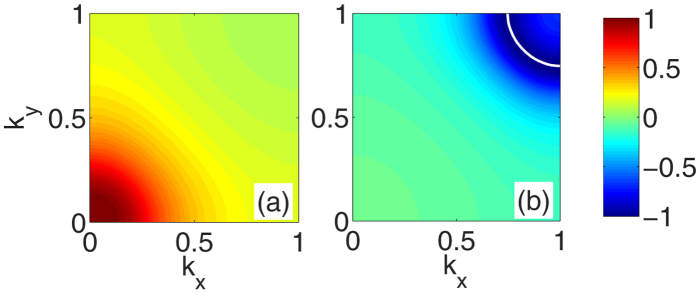
The momentum dependence of eigenvectors at the lowest Matsubara frequency (*πT*) for 〈*n*〉 = 1.05 and *U* = 2.4*t* at *t*_⊥_ = 3.8*t*. The eigenvector is normalized to its maximum value. The momentum dependence for the incipient hole band is shown in panel (a) and for the electron band is shown in panel (b) with the electronlike Fermi surface.

The momentum dependence of the superconducting gap function Δ(*k*, *ω* = *πT*) ≡ Φ(*k*, *πT*)/*Z*(*k*, *πT*) is shown in Fig. 3. This is an *A*_1*g*_ (s_±_) state in which the sign of Δ changes between *k*_*z*_ = 0 (bonding) and *k*_*z*_ = *π* (antibonding) bands. One can see that the magnitudes of the two gaps Δ(*k*_*x*_, *k*_*y*_, *k*_*z*_ = 0) and Δ(*k*_*x*_, *k*_*y*_, *k*_*z*_ = *π*) are comparable even though the hole band is below the Fermi energy.

In order to understand the peak in *T*_*c*_ that occurs as the hole band drops below the Fermi energy, it is useful to separately examine the dependence of *T*_*c*_ on the changes in 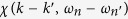 and 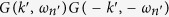 that occur as the *T*_*c*_ at which the eigenvalue of Eq. (5) goes to 1 as a functional of *χ* and the pair propagator *GG*. We can calculate the variation in *T*_*c*_ due to the change in *χ* with *GG* unchanged when *t*_⊥_ increases by Δ*t*_⊥_,


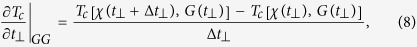


and the variation due to the change in pair propagator *GG* when *χ* is unchanged and *t*_⊥_ increases by Δ*t*_⊥_,


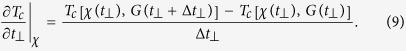


**Figure 4:**
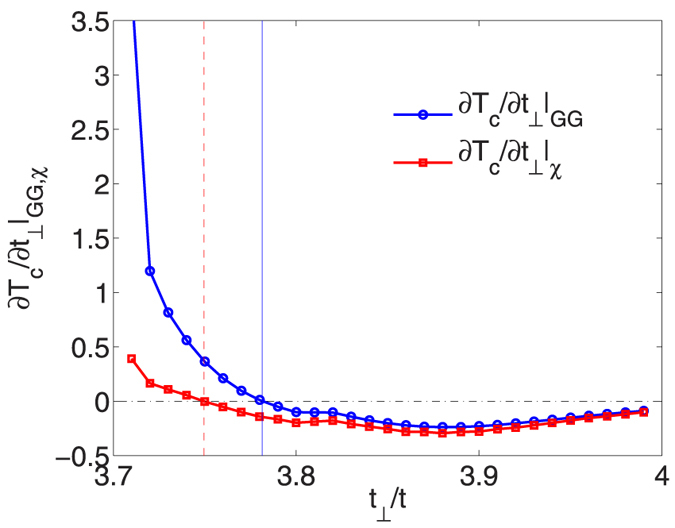
The variation of *T*_*c*_ with changes in *χ* (solid blue curve) and the pairfield propagators *GG* (dashed red curve) versus *t*_⊥_/*t*. Here one sees that initially as *t*_⊥_/*t* increases beyond 3.7 and the SDW order is suppressed, both the change in *χ* and the change in *GG* lead to an increase in *T*_*c*_. Then, as *t*_⊥_/*t* increases further and the hole band drops deeper below the Fermi energy, the changes in both *χ* and *GG* lead to a reduction in *T*_*c*_.

**Figure 5:**
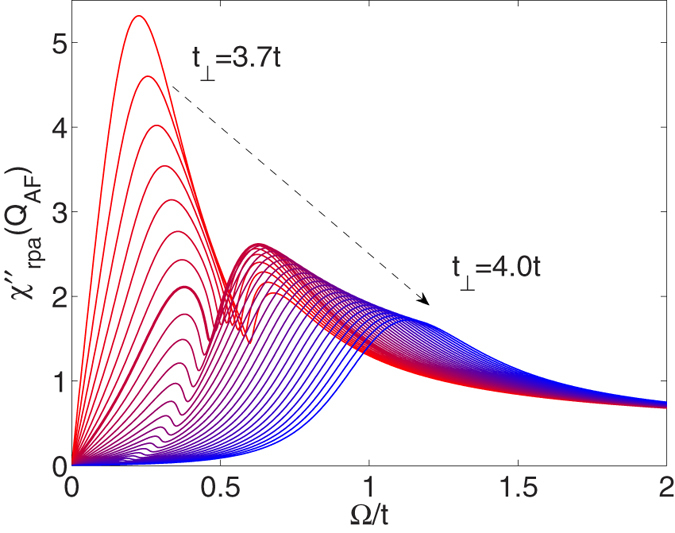
The imaginary part of RPA *χ*(*π*, *π*, *π*, Ω) at *T* = 0.1*t* versus Ω for *U* = 2.4*t* for different values of *t*_⊥_, which continuously increases in units of 0.01*t* from *t*_⊥_ = 3.7*t* (red curve)to *t*_⊥_ = 4*t* (blue curve). The thick line represents the value of *t*_⊥_ = 3.77*t* corresponding to the maximum value of *T*_*c*_. As *t*_⊥_ increases and the hole band drops below the Fermi energy, the spin fluctuation spectral weight is shifted to higher frequencies, of order 5 to 10*T*_*c*_, where it is more effective for pairing. However, as *t*_⊥_ increases further and the spectral weight moves to still higher frequencies, the strength of the pairing decreases and this combined with the suppression of the pair propagator *GG* leads to a rapid decrease of *T*_*c*_.

We set Δ*t*_⊥_ = 0.01*t*. The results of the calculation are shown in Fig. 4. Here one sees that the initial increase in *T*_*c*_ arises from both the changes in *χ* and *GG*. The latter effect is associated with an increase in the quasi-particle spectral weight *Z*^−1^(*k*, *ω*) on the electron Fermi surface that occurs as the hole band drops below the Fermi energy. This increase in the quasi-particle spectral weight initially ameliorates the decrease in *T*_*c*_ resulting from the submergence of the hole band. The initial positive contribution associated with the variation in *χ* reflects the change in the frequency structure of the spin-fluctuations. As the hole band drops below the Fermi energy, a gap opens in the low energy *q*_*z*_ = *π* spin fluctuation spectrum and spectral weight is transfered to higher energies as shown in Fig. 5, which leads to stronger pairing^20^. The ultimate decrease in *T*_*c*_ is due to the decrease of the pair propagator 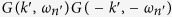 as *t*_⊥_ increases and the hole band drops further below the Fermi energy, as well as the decreasing strength of the spin-fluctuations.

## Discussion

To conclude, we have studied a two-layer Hubbard model with parameters chosen so that a SDW instability is suppressed by a Lifshitz transition in which the hole band at the Γ point drops below the Fermi energy. Here, we have kept the site filling fixed and varied the interlayer hopping to tune the system through the Lifshitz point. For a physical system this might by obtained via strain^11^. Following the suppression of the SDW order, we find the onset of an s_±_ superconducting state whose transition temperature *T*_*c*_ initially increases as the system is pushed beyond the Lifshitz point by further increasing *t*_⊥_/*t*. We find that this increase in *T*_*c*_ is associated with both an increase in the quasi-particle spectral weight and an increase in the strength of the pairing interaction, which are related to the incipient hole band and the resulting change in the spectral distribution of the spin-fluctuations. We find that the gap function on the incipient hole band is similar in magnitude but of the opposite sign to that on the electron band which crosses the Fermi surface.

## Additional Information

**How to cite this article**: Mishra, V. *et al*. s_±_ pairing near a Lifshitz transition. *Sci. Rep*. **6**, 32078; doi: 10.1038/srep32078 (2016).
